# Toxicology of chemically modified graphene-based materials for medical application

**DOI:** 10.1007/s00204-014-1361-0

**Published:** 2014-09-19

**Authors:** Toktam Nezakati, Brian G. Cousins, Alexander M. Seifalian

**Affiliations:** 1 UCL Centre for Nanotechnology and Regeneration Medicine, Division of Surgery and Interventional Science, University College London, London, UK; 2 Royal Free London NHS Foundation Trust, London, UK

**Keywords:** Biocompatibility, Graphene, Graphene oxide, In vitro, In vivo, Toxicity

## Abstract

This review article aims to provide an overview of chemically modified graphene, and graphene oxide (GO), and their impact on toxicology when present in biological systems. Graphene is one of the most promising nanomaterials due to unique physicochemical properties including enhanced optical, thermal, and electrically conductive behavior in addition to mechanical strength and high surface-to-volume ratio. Graphene-based nanomaterials have received much attention over the last 5 years in the biomedical field ranging from their use as polymeric conduits for nerve regeneration, carriers for targeted drug delivery and in the treatment of cancer via photo-thermal therapy. Both in vitro and in vivo biological studies of graphene-based nanomaterials help understand their relative toxicity and biocompatibility when used for biomedical applications. Several studies investigating important material properties such as surface charge, concentration, shape, size, structural defects, and chemical functional groups relate to their safety profile and influence cyto- and geno-toxicology. In this review, we highlight the most recent studies of graphene-based nanomaterials and outline their unique properties, which determine their interactions under a range of environmental conditions. The advent of graphene technology has led to many promising new opportunities for future applications in the field of electronics, biotechnology, and nanomedicine to aid in the diagnosis and treatment of a variety of debilitating diseases.

## Introduction

There is only a relatively small contribution regarding the safety profile and toxicology data in the literature on graphene-based materials outlining their interactions in biological systems with cells and tissues. Over the last 5 years alone, over 424 publications and cited articles relate to graphene toxicology, which has increased to 1,015 publication by 2009 to approximately 3,753 in 2013, whereby the vast majority focus on the physical and material properties of graphene and is a subject of intensive research (Liao et al. 2011; Hu et al. 2011). The physicochemical interaction of graphene, and their use in biological systems, is perhaps one of the newest and fastest growth areas of carbon-based nanomaterials research. Much study in this area is inspired by the myriad of possibilities of many useful biomedical applications relating to their unique properties and to address healthcare concerns relating to nanotoxicology (Liu et al. 2008; Chang et al. 2011a). There has been an intensive focus over the last 10 years in the application of carbon-based nanomaterials such as charcoal, graphite, fullerene, single-wall carbon nanotubes (SWCNTs), multi-wall carbon nanotubes (MWCNTs), and graphene. This is due to the exploitation of their unique properties, such as enhanced electrical, thermal, mechanical, and optical properties, which provides a range of different application areas from advanced electronics and imaging to biomaterials and biological sensors for diagnostic use. However, a major concern, involving graphene-based materials, is that there is a limited knowledge relating to their environmental toxicity and biological safety profile. The UK government body, the Medicines and Healthcare Products Regulatory Agency (MHRA), and the US Food and Drug Administration (FDA) are now reviewing all forms of graphene and functionalized graphene oxide (GO) due to their poor solubility, high agglomeration, long-term retention, and relatively long circulation time in the blood (Begum et al. 2011). Extensive testing is now deemed essential for graphene-based materials both for now and in the near future to assess their biological safety profile, which is dependent upon different physicochemical factors relating to their surface chemistry, charge, size, shape, and relative concentration. Yet still there are many unresolved issues, which remain and need to be clarified before their eventual use for healthcare applications can be fully realized. The biocompatibility and toxicity behavior of graphene-based material in biological systems gives rise to many important fundamental issues that require significant attention, and numerous studies are now needed to fill the knowledge gap before being considered as truly ‘safe’ for human use.

## Graphene structure and related properties

**Fig. 1:**
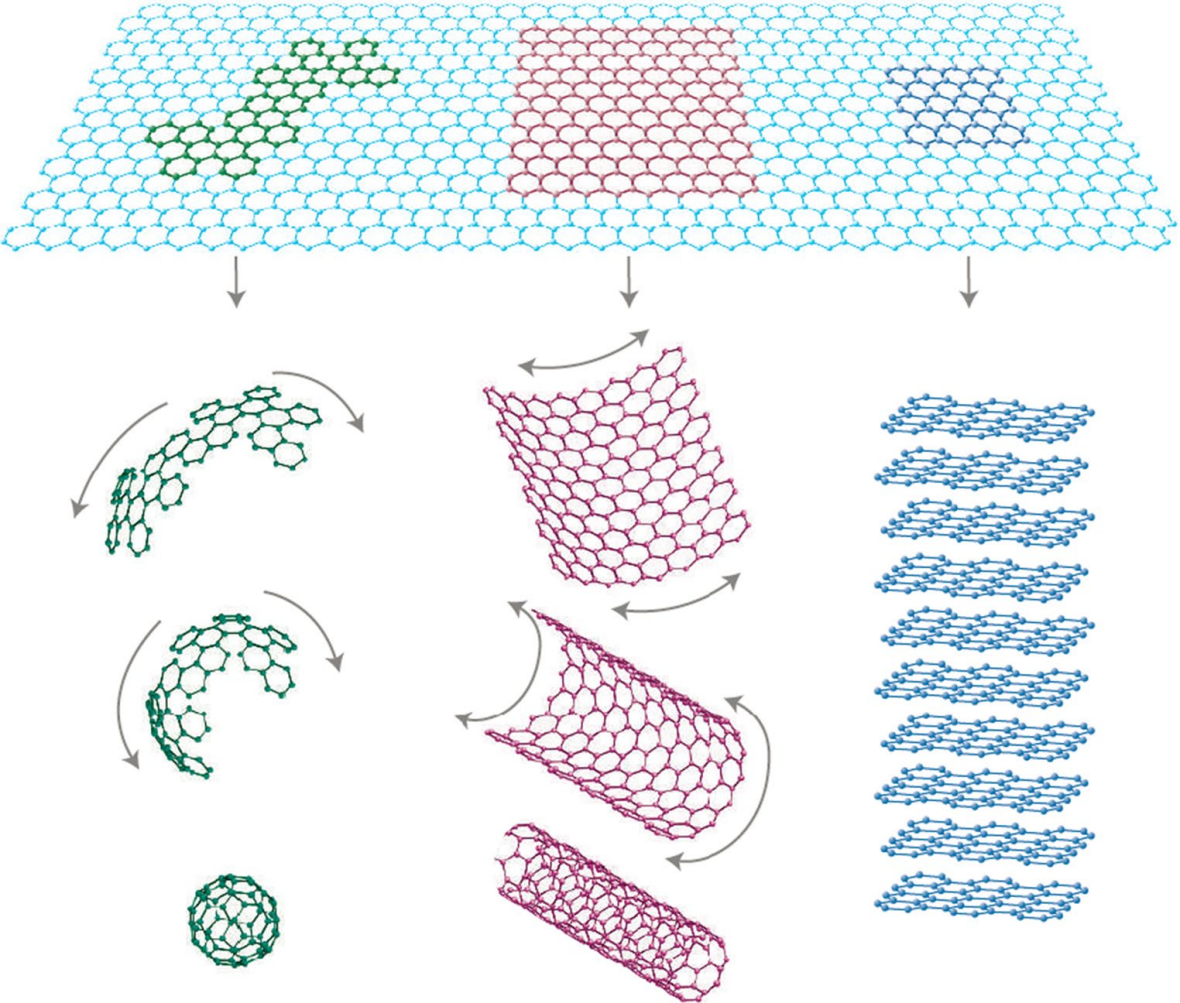
Graphene is a 2D building material for allotropes of carbon nanomaterials. It can be wrapped up into 0D buckyball, rolled into a 1D nanotube, or stacked into 3D graphite (Geim and Novoselov 2007)

Graphene is composed of single-carbon atoms forming a sheet of close-packed hexagonal array of SP^2^ hybridized bonds and can be considered as large aromatic molecule. As such, they have attracted a significant amount of attention in recent times, especially in various areas of biophysics and biotechnological applications (Mao et al. 2013b). The two-dimensional, graphene flat sheets can be formed into different geometries, which can be wrapped into spherical structures (0D fullerenes, C_20_, C_40_, C_60_), rolled into 1D structures as a single-sheet CNTs, or stacked into 3D-layered structures such as graphite (Fig. 1) (Geim and Novoselov 2007). This is due to their exceptional material properties giving rise to unique chemical, electrical and thermal conductivity (~5,000 Wm^−1^ K^−1^), mechanical, optical transmittance (~97.7 %), structural, and thermal behavior, and has shown great promise for many application areas relating to electronics, semiconductor fabrication, and the biomedical industry (Zhu et al. 2010; Compton and Nguyen 2010; Rao et al. 2009). Graphene has a number of fascinating physical characteristics such as the highest surface area (~2,600 m^2^/g) (Li et al. 2008) and a relatively high Young’s modulus (<1 TPa) among all known materials (Lee et al. 2008), and capable of mass production through a number of chemical manufacturing and material processing such as non-covalent and covalent surface modification using surfactants, and biofunctionalization to exploit their unique properties (Shao et al. 2010). Moreover, graphene consists of a layer of π-conjugated systems usually involving six-atom rings. This planar structure offers an excellent capability to interact with a variety of aromatic compounds through *π*–*π* stacking interactions in the manufacture of nanocomposite materials and in the immobilisation of biomolecules such as peptides, antibodies, and other therapeutic agents (Boehm 1986; Wintterlin and Bocquet 2009; Van Bommel et al. 1975; Lu et al. 1999a, b; Novoselov et al. 2004). Therefore, graphene has generated great interest in the field of nanomedicine and has been successfully applied in biosensing applications via targeted and selective delivery (Shao et al. 2010; Akhavan et al. 2012b), bioimaging, cell culture, cancer detection, gene delivery (Boehm 1986; Wintterlin and Bocquet 2009; Van Bommel et al. 1975; Lu et al. 1999a, b; Novoselov et al. 2004; Berger et al. 2004; Li et al. 2009; Stankovich et al. 2006), disease diagnosis (Mohanty and Berry 2008), anti-bacterial compounds (Akhavan and Ghaderi 2009, 2010, 2012; Hu et al. 2010; Ma et al. 2011; Akhavan et al. 2011), anti-viral materials (Akhavan et al. 2012c), photo-thermal therapy (Yang et al. 2012b; Zhang et al. 2011a; Akhavan et al. 2012a), drug delivery (Sun et al. 2008; Liu et al. 2008, Li et al. 2011; Zhang et al. 2010a), and tissue engineering applications (Park et al. 2010; Agarwal et al. 2010; Heo et al. 2011). Therefore, all of their interesting material properties propel graphene from the research laboratory to real-life biological and clinical applications and show great potential for further exploitation and use within the biomedical industry ready for clinical use.

## Graphene preparation and manufacture

**Fig. 2:**
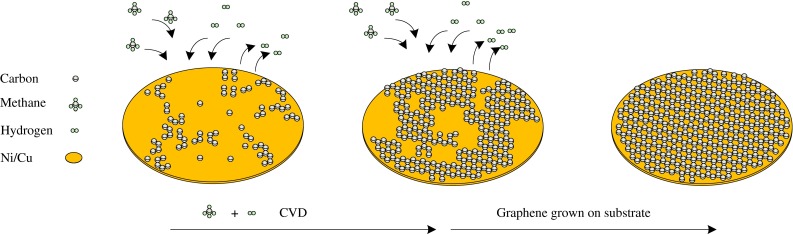
Bottom-up fabrication, by chemical vapor deposition (CVD)

**Fig. 3:**
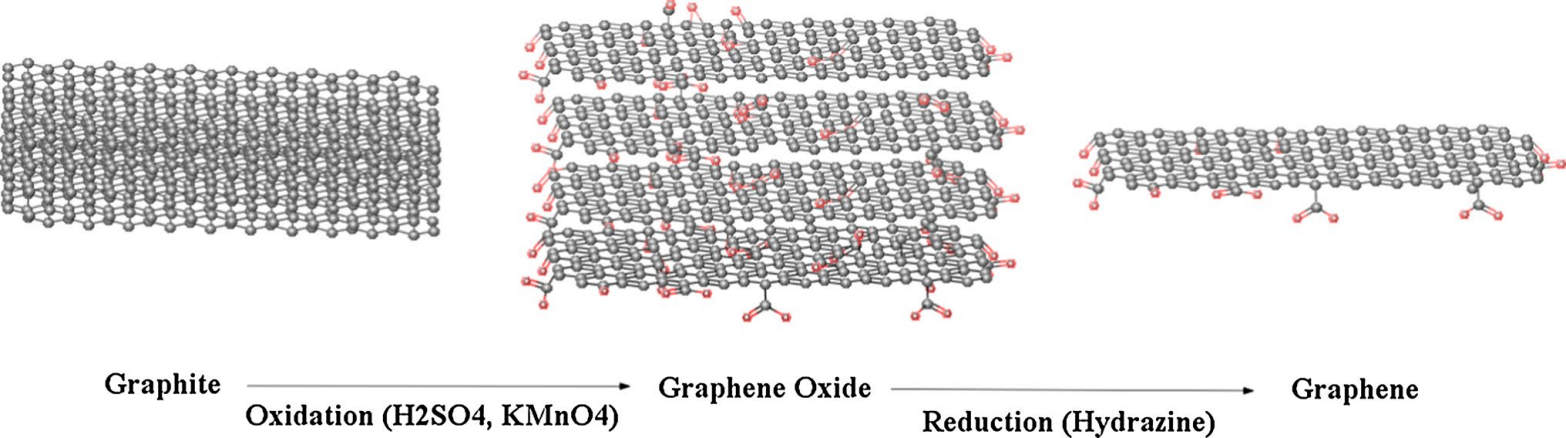
Top-down fabrication, solution based on Hummer’s method using ultrasonication

The preparation of graphene can be divided in two main categories: (1) bottom-up and (2) top-down fabrication techniques. Bottom-up fabrication is achieved using several methods to prepare high-quality graphene such as chemical vapor deposition (CVD). These methods produce highly crystalline graphene, but are not suitable for mass production (Graphene et al. 2010; Kim et al. 2011). For example, CVD is a method which opens up scalable and transparent high-quality graphene in ultra-high vacuum (UHV) conditions (10^−4^–10^−6^ pa) at high temperature (1,000 °C) using gasses such as methane –CH_4 (g)_ as a carbon source as highlighted in Fig. 2. The CVD process revolves around a piece of copper (Cu) foil on silicon substrate, which is used as catalyst, which graphene is able to grow as a fibrous ‘mat’-like material. At very high temperatures in an extreme clean, UHV chamber (or environment), carbon from CH_4_ forms graphene on top of the Cu or nickel (Ni) foil (Fig. 2). Current methods are derived from chemical modification, and functionalized GO and reduced graphene oxide (rGO) within the top-down category are achieved through chemical exfoliation (Novoselov et al. 2012; Dreyer et al. 2010). Chemical exfoliation, described by Schafhaeutl, in the 1940s is a method, which uses a wide range of chemicals such as acid or alkali metals (e.g., potassium), fluoride salts of various types, and transition metals (e.g., iron, nickel), to obtain GO (Dreyer et al. 2010). Nineteen years after Schafhaeutl described this method, British chemist, Broid, used a chemical exfoliation process to manufacture GO. This method can characterize the molecular weight of graphite by using acids (e.g., sulfuric and nitric), as well as oxidants, such as potassium chlorate (KClO_3_). Further exfoliation with ultrasonication, thermal or energetic conditions help to oxidize stacked layers of hexagonally arranged carbon atoms that are bonded together with an inter-planar force to obtain graphene layers. The use of this method led to the formation and production of single-layer-reduced GO (Dreyer et al. 2010). Top-down fabrication involves a chemical reduction based on Hummer’s method (Hummers and Offeman 1958), and chemical oxidation of graphite followed by ultrasonication is highlighted in Fig. 3.

## Use of graphene in biomedical applications

Owing to graphene’s low level of toxicity, the lethal dose (LD50) of graphite has been reported as 2 g/per kilo (Sebastian 2012); the following sections outline some of the most promising application areas, use of graphene for enhanced imaging, diagnostics and therapeutic applications in nanomedicine, and their use as novel materials for improved medical devices via improvements in their mechanical properties, and photosensitivity has received considerable attention, along with their health and safety and regulatory concerns (Yang et al. 2010, 2011a, b).

### Drug delivery applications

The treatment of cancer represents a global challenge to public health care and is a leading cause of over 7 million deaths worldwide annually (Wood 2013; Boyle and Levin 2008). One significant and area of great importance in the treatment of cancer is the application of chemotherapy. This approach has proved successful in the treatment of various cancers, such as acute promyelocytic leukemia (Jing 2001; Chen et al. 1997), lung (Carney et al. 1983; Umezawa et al. 1966; Kouranos et al. 2011), head and neck cancers. However, the lack of therapeutic efficacy confines such clinical applications due to drug resistivity, low efficiency of cellular uptake, and high proportion of side effects, such as liver and kidney damage (Calvert et al. 1989; Kintzel and Dorrt 1995), hair loss (Jaracz et al. 2005; Narang and Varia 2011), nausea and cardiac toxicity (Chithrani et al. 2009; Geiger et al. 2010; Voortman and Giaccone 2006). Therefore, novel materials with minimal side effects, low toxicity, and high efficiency of targeted drug delivery enhance the bioavailability for chemotherapy, which is an area of increasing research interest (Abou-jawde et al. 2003; Manuscript 2009). Lung cancer is the primary cause of death for all known cancers worldwide (Deaths 2011; Jemal et al. 2011), and due to the size and distribution, cyto-reductive surgery is rarely a viable treatment option. Chemotherapy based on cytotoxic drugs kills cancer cells, which is the main popular approach for treatment of lung cancer. However, the lack of targeting specificity leads to severe side effects such as hemorrhage (Manuscript and Factors 2008). More effective localized delivery can lead to substantial improvements in curative and therapeutic modes of action not only for chemical-based treatments, but for MRI gene delivery including contrast enhancers and radiation sensitizers. In addition, the precise diagnosis and therapy are difficult in most cases for the limited options available (Shi et al. 2013a). Therefore, enormous endeavor in biomedical research has been dedicated to developing new approaches for early-stage detection, diagnosis, and therapy of cancer, which is now commonly referred to as ‘theranostics’ (Mura and Couvreur 2012). Driven by an unmet clinical need, highly integrated drug delivery nanocarriers rely for simultaneous imaging and therapy are currently being evaluated (Huang et al. 2012; Melancon et al. 2011; Liang 2011; Jokerst and Gambhir 2011). Graphene and its derivatives, such as GO, reduced GO, and GO nanocomposites, are some of the more well-known examples (Feng and Liu 2011). Externally controlled non-invasive drugs with reliable remote sensing and repeatable ‘on’ and ‘off’ molecular switches to control drug release have recently been receiving attention (Thomas et al. 2010). This method consists of drug-releasing technology via an external stimulus to induce carrier responsive and material properties. The external stimulus is usually derived from polarized or infrared (IR) light (Yavuz et al. 2009; Sherlock et al. 2012; Lu et al. 2008), magnetic field strength (Hoare et al. 2009; Thomas et al. 2010), ultrasound (Hu and Zhou 2014), and radio frequency-induced drug delivery (Santini et al. 1999; Grayson et al. 2003).

### Photo-thermal therapy (PTT)

**Fig. 4:**
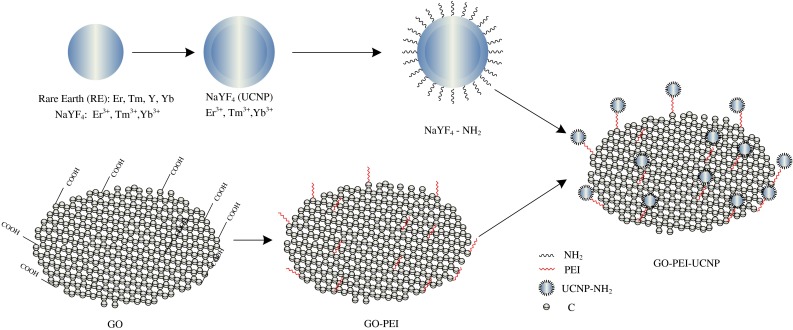
Schematic diagram of the procedure for GO–PEI–UNCP: Numbers of core-shell structured UCNPs covalently grafted with GO through polyethylenimine for advanced imaging, drug delivery, and photo-thermal therapy

Photo-thermal therapy (PTT) converts light or optical energy to heat by absorption of a range of nanomaterial (e.g., silica-coated gold nanoparticles), leading to the thermal ablation resulting in the death of cancer cells. In recent years, PTT as a minimally invasive, controllable, and highly efficient treatment method has drawn widespread attention in the treatment of cancer. A large number of research groups have developed various light-absorbing nanomaterials as PTT agents (Huang et al. 2006; Chen et al. 2007; Yavuz et al. 2009; Wu et al. 2010; Dong et al. 2011; Tian et al. 2011; Cheng et al. 2011, 2012; Yang et al. 2010, 2012b, c; Moon et al. 2009; Liu et al. 2011; Wang et al. 2011, 2012), all with absorbance values in the near-infrared (NIR) region (560–760 nm), which is the region ideal for controlling interactions with biological tissues. Despite the great promise of PTT in cancer treatment using nanomaterials, the development of a new generation of PTT agents with enhanced NIR absorption and multiple functions to realize imaging-guided highly effective cancer therapy still merits further effort. Carbon-based nanomaterials, such as CNTs, carbon nanohorns, and graphene, are being extensively studied as potential PTT agents (Moon et al. 2009; Liu et al. 2011; Wang et al. 2011, 2012; Yang et al. 2010, 2012b). Besides inorganic materials, organic nanoparticles, such as polypyrrole and other light-absorbing conductive polymers, have also shown potential in PTT cancer ablation in a few recent studies (Cheng et al. 2012; Yang et al. 2012c; Chen et al. 2012c; Zha et al. 2013). Nanoparticle-based NIR-PTT provides an encouraging remedy and strategy for efficient tumor ablation with minimum injury to the surrounding tissues. Up-conversion of nanoparticles (UCNPs) is a further approach to PTT. As an example, UNCP, water-dispersible nanocrystals, which is fluorophores and magnetic nanoparticles, whereby ferric oxide (Fe_3_O_4_) is reacted with polyethylenimine-modified GO (PEI-GO) acting as a nanocarrier attached to the nanocrystals to yield PEI-GO–nanocrystal (Yan et al. 2013). PEI-GO–UCNP is able to load water-insoluble anticancer drugs, such as doxorubicin (DOX), with a superior loading capacity of 100 wt%, through hydrophobic, *π*–*π* stacking interaction between PEI-GO–UCNP, and an aromatic drug highlighted in Fig. 4. Chemotherapy and PTT when used in combination have been proven to reduce drug resistance, and to be an effective strategy to improve the cancer therapy efficacy (Tang et al. 2010; Tang and Mcgoron 2009; Hauck et al. 2008; Lee et al. 2010). In contrast, undesired damage to normal tissues may be caused by non-specific, untargeted drug delivery and heat supplied to the tumor area. Moreover, recent studies suggest that graphene possesses a higher photo-thermal sensitivity than CNTs, and is more effective in PTT in the treatment of cancer (Markovic et al. 2011; Yang et al. 2010, 2012a; Tian et al. 2011).

### Nerve repair and regeneration

There is currently an unmet clinical need for biocompatible and conductive materials used for neurological applications, which are crucial in the development of next generation of chronic (long-term) implants used in the peripheral and central nervous system (CNS). Nanoparticles incorporated into polymeric conduits, acting as fillers, such as, graphene, CNTs, and fullerene, can become one possible solution in the production of conducting materials, which are necessary for stimulating cell growth, and delivery of therapeutic agents. Identification of neural stem cell differentiation is an essential stage for the practical application of stem cell technology in regenerative medicine. Cell differentiation and monitoring is incredibly important for the application of neural stem cells (NSCs) in the treatment of neurodegenerative disease such as Alzheimer’s (Steindler and Okun 2012), Parkinson’s disease (Steindler and Okun 2012; Daadi et al. 2012; Xie et al. 2012), and also, traumatic spinal cord injury (Li et al. 2012; Donnelly et al. 2012). Many conventional tools have been used to detect the differentiation potential of NSCs, as well as to distinguish the undifferentiated NSCs from differentiated neuronal and glial cells (Danova-alt et al. 2012; Ganat et al. 2012; Piao et al. 2012; Buján et al. 2005; Xu et al. 2012).

## Risk assessments of graphene-based nanomaterials

**Fig. 5:**
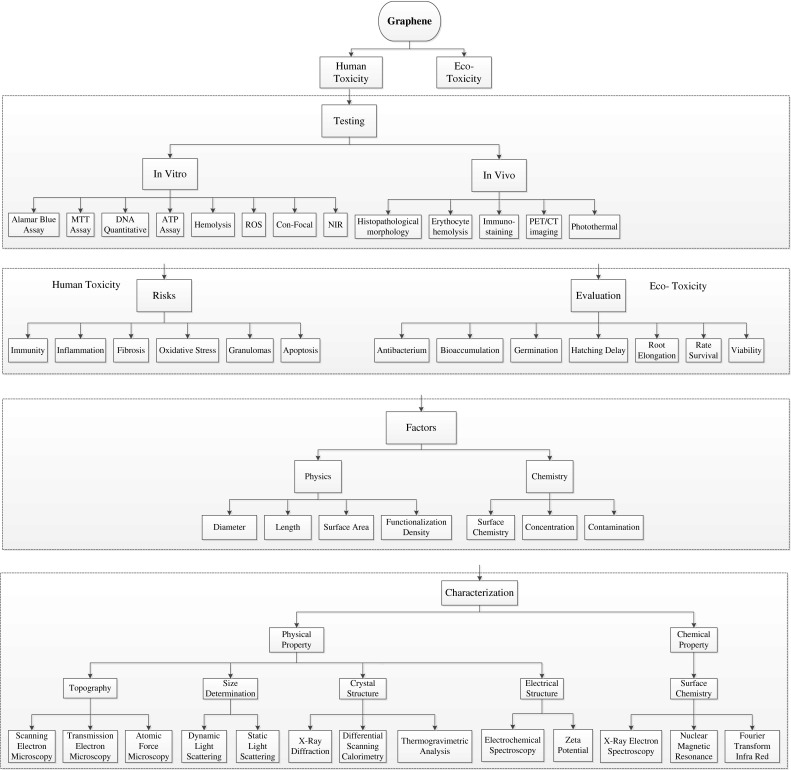
Toxicology of graphene: An overview on the principal components of graphene toxicity

**Fig. 6:**
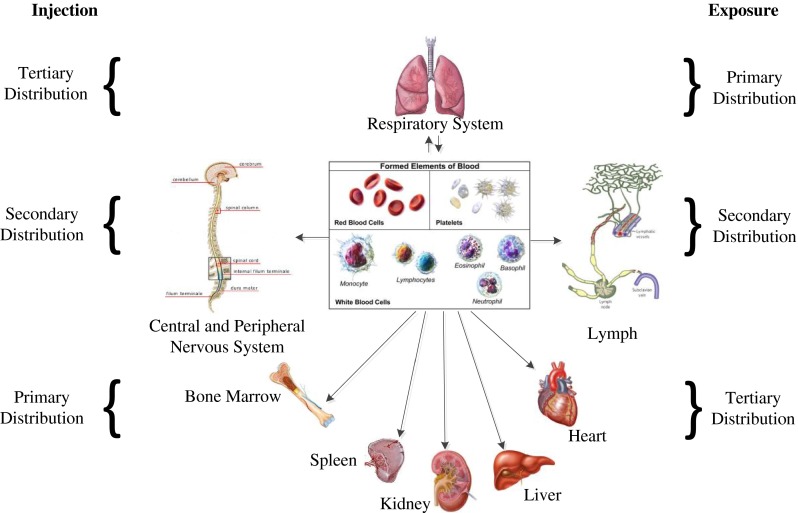
Distribution of graphene in the body

According to the elemental composition of carbon atoms arranged within the graphene layer or as discrete nanoparticles, knowledge of influential factors such as their surface chemistry (energy, charge, and wettability), morphology, geometry and aggregation behavior and solubility will influence the particle distribution within the surrounding environment. Nanoparticles when present in physiological fluids such as plasma or serum redistribute themselves throughout the host tissues and are transported to the liver, lungs, spleen, heart, kidney, and bone marrow due to their material and surface properties, and how they interact as a consequence of proteins that adsorb on to the surface can cause nanoparticle aggregation and cell uptake (Gajewicz et al. 2012). Figure 5 illustrates the toxicology overview on the principal components of graphene, the tests that are required, risk factors, and their eventual characterization, highlighting the need for standardization for testing this class of material. Investigation via in vitro studies has shown that the indirect contact with nanomaterials with mammalian cells causes cytotoxic reactions, such as oxidant release via reactive oxygen species (ROS) and stress followed by cytokine release and inflammation, which is primarily in response to ROS (Nel 2005; Nel et al. 2006; Oberdörster et al. 2005), cell damage and lipid peroxidation of cellular membranes (Nel et al. 2006; Oberdörster et al. 2005; Panessa-Warren et al. 2006, 2008). Such events are known to cause changes in gene expression, which involve irregular signaling cues influencing cell fate resulting in further inflammation (Cui et al. 2005). The toxicity profile of graphene and GO nanoparticles remains elusive, since their characterization, bulk and chemical composition are very similar at the nanometer length scale. Figure 6 shows the potential distribution of graphene, highlighting the target organs and systems and distribution throughout the human body (Zhao and Liu 2012). A number of previous studies report that pristine graphene or GO without further surface modification causes severe pulmonary distress after inhalation causing excessive inflammation (Duch et al. 2011). Intravenous (i.v.) injection of functionalized graphene into mice is known to accumulate in the lung resulting in pulmonary edema and granuloma formation (Wang et al. 2010; Zhang et al. 2011a, b). Furthermore, surface-functionalized graphene with improved hydrophilicity and better stability in the physiological environment appears to be far less toxic (Singh et al. 2012; Yang et al. 2011a, b). In Fig. 6, graphene can easily enter into the lungs via the respiratory system and later distribute themselves in the circulatory system via the blood and lymph fluid (Fig. 6). Further investigation into the distribution of graphene has found that the materials can penetrate into the tissues of the heart, spleen, kidney, bone marrow, and liver. The major risks to health care associated with the manufacture of carbon-based nanomaterials are to the eyes and the lungs, and can cause substantial irritation and inflammation (Kayat et al. 2011).

### Exposure assessment of graphene

Due to the ever expanding applications of nanotechnology, human and environmental exposures to graphene-based nanomaterials are likely to increase both now and in the near future (Seabra et al. 2014). It is still not clear how to establish the precise mechanism or precise laboratory-based test for determining the potential toxicity of nanoparticles. In the development of novel nanomaterials, considerable effort is needed to perform as broader risk characterization as possible (Worth 2010). Currently, one of the general market risk assessment methods is exposure methods to determine whether an exposed population and dependent exposures factors like frequency, magnitude, and duration have a cumulative effect. Other methods rely upon hazard assessments like identification and characterization of known hazards. Environmental factors also determine the exposure levels and distribution of the chemicals in the natural environment such as the levels present in the soil, sediment, water, and air, and any analytical measures as to the conversion or deterioration of chemicals, and the media containing the specific agents movement throughout the environment. The specific risk of the materials can be estimated, processed, and individualized within any given systems, while the hazard assessments will qualitatively identify the nature of adverse effects and quantitatively define dose effects and dose–response relationships. All of the most recent reviews focus on the most important aspects of the risk assessment of graphene-based nanomaterials, which determine the extent to which the range of concentration of a given chemical released into the environment (e.g., level of exposure) overlaps in time and space with those that are toxic (e.g., hazardous to health) in a range of selected organisms in a given populations within the ecosystem. Considering factors such as exposure and medical treatments and due to the different variables, it is required to be very precise to consider the best graphene based nanomaterials. Such variables are often complex and are complicated further by established ecosystems, and the numerous inter-relationships, and multivariant pathways in the distribution of chemicals and nanoparticles in the natural environment (Toxicology 1990).

### Hazard assessment of carbon-based nanomaterials

Hazard assurance that a chemical or nanoparticle can show up in the tissue of the host and any other living organism is usually dependent upon the frequency, concentration, and duration of exposure of the materials due to factors including the magnitude, extent, and duration to cause toxic side effects. Individual assessments and combinatorial hazard assessments are now required, and are in great demand to identify and characterize the causative agents. This is primary due to exposure (as described previously), as a result of environment toxicity or in vitro or in vivo medical treatments using graphene either as an implantable device or as drug delivery carrier. Animal experiments are performed according to policy guidelines standardized by the Organization for Economic Co-operation and Development (OECD) in the UK, and are known to determine common toxic characteristics of a broad range of materials and chemicals. This includes eco-toxicity or animal and human toxicity.

#### Animal and human toxicity

Toxicology studies are now becoming more advanced in small- and large-scale animal studies in vivo and human cell lines in vitro. Direct hazard assessments and current methodology for studying nanomaterials help to reveal gaps in the knowledge and deficiency of current assessments. For example, the lethal dose (LD50) of graphite, CNTs, and fullerenes reported as 2 g/Kg (Sebastian 2012), 2 mg/Kg (Ragot et al. 2010), and 1.2 g/Kg (Da Ros et al. 2001), respectively, in animals. It is essential to employ traditional risk assessments and control of substances hazardous to health (COSHH) procedures when dealing with engineered nanoparticles, as information from toxicology studies is often deficient for broad risk assessments to be made with regard to nanomaterials based on carbon due to their vast heterogeneity. The most common cytotoxicity assays to evaluate toxicity of graphene-related materials are apoptosis assay’s such as caspase-3,7 assays to measure cell death, cell adhesion and morphology, cytokine detection, hemocompatibility, hemolysis; lactate dehydrogenase (LDH) assay to assess membrane integrity; methyl thiazolyl tetrazolium (MTT) assay as a measure of metabolic activity, platelet activation, ROS generation, and genotoxicity (Bitounis et al. 2013; Vallabani et al. 2011; Zhang et al. 2010b; Schinwald et al. 2012; Chang et al. 2011b; Akhavan et al. 2012d; Liao et al. 2011; Sasidharan et al. 2012; Seabra et al. 2014). Since there is a very close relationship between mutation and damage to DNA, genotoxicity assays are considered as an early and important indicator of toxicity, which may lead to cancer and tumor development (Agemy et al. 2010). The MTT is perhaps the most popular assay among the cell-based assays, and used for cytotoxicity and cell viability when characterizing nanomaterials. In addition, this method using CNTs as the test substrate has been reported to be problematic as a consequence of using graphene-based materials (Bitounis et al. 2013; Liao et al. 2011; Seabra et al. 2014). Therefore, the cell proliferation assay (WST-8) is a preferred method instead of MTT (Liao et al. 2011). Thus, the most appropriate cytotoxic assays must be used to evaluate the toxicity of graphene-based materials to avoid false data. Graphene-based nanomaterials’ in vivo studies are mostly based on the evaluation of tissue distribution (bioaccumulation) and excretion from the body. The most common animal model used to evaluate in vivo toxicity of graphene-based materials is the Zebrafish model (Fako and Furgeson 2009). The route of administration should be considered as an important parameter that impacts the toxicity of nanomaterials (Yang et al. 2013a). Based on the recent literature, it is clear that due to the increase in the importance of graphene-based materials, meticulous and accurate in vitro and in vivo studies and accurate testing models of toxicity of the growing graphene family are required and are now in great demand.

#### Eco-toxicity of graphene-based nanomaterials

Above all other living creatures on the earth, continual evolution brought intelligence to mankind. Therefore, it is necessary that the study of eco-toxicological hazards and their assessments are of considerable importance. In toxicology, the focus is on human as a species, but in ecotoxicology the focus is broadened significantly, regarding the safety and well-being of millions of other common and rare species. So environment factors could put human needs at risk due to indirectly addressing the safety of harmful chemicals, which can degrade or even destroy ecosystem (Toxicology 1990). The terrestrial environment is similar ecologically to aquatic environments, because living organisms are often intertwined and share a common food chain associated with their natural environment. Two of the well-known organized biological communities are the plant and animal kingdom. The plants are almost affected directly by the level of exposure and the presence of pollutants in the air and in rain fall. However, animals and sometimes humans can become contaminated with heavy metals by uptake toxic compounds through the food chain, e.g., mercury compounds. In the aquatic ecosystem, there is direct contact between the pelagic fauna and flora with the chemicals suspended or dissolved in water. In contrast, the food chain is considered as indirect contamination or deemed much slower than direct contamination. Both ecosystems can be contaminated by direct exposure or ingestion of the particles. The quantities of chemical substances, which are carried by different media, such as in the air, soil, or water, are often variable in nature. Furthermore, in different media the bioavailability and dynamics are very different. Eventually, such variability and different approaches applied for terrestrial and aquatic organisms raise substantial concerns in terms of accurately determining the levels of toxicity when comparing similar ecosystems.

## Toxicity of functionalized graphene oxide and functionalized graphene

There are very few reports available on the toxicity of graphene in vitro (Liu et al. 2009; Singh et al. 2011; Sasidharan et al. 2011) and in vivo (Chang et al. 2011a; Yang et al. 2011a, b) compared to carbon nanostructures, fullerenes (Service 2003), and CNTs (Nakamura and Isobe 2003; Lacerda et al. 2006). The main parameters affecting cytotoxicity of this class of nanomaterial including graphene (Wang et al. 2010), GO (Hu et al. 2010), CNTs (Chang et al. 2011a; Lam et al. 2006), gold and silver nanoparticles (Lee et al. 2011) in vitro and in vivo are concentration, shape, size, surface charge, energy, and wettability. Also, in vitro studies could be divided in two discrete sections: (1) cytotoxicity and (2) genotoxicity. Extensive studies have been performed relating to in vitro cytotoxicity of GO over the last 5 years. However, the investigation of new areas of concern relating to genotoxicity of nanomaterials is an important research theme, as there is a close correlation between DNA damage, mutation, and the formation of cancers (Agemy et al. 2010). There is insufficient research carried out on the genotoxicity of graphene-based materials at present and warrants much further investigation.

### Toxicity of functionalized GO

GO is water-soluble nanomaterial and has been investigated extensively as a material for industrial applications for electronics and use in biomedical engineering. This is due to the large vacancy of planar surface area for efficient filling of aromatic drug molecules through *π*–*π* stacking interactions, and carboxyl (–COOH), epoxy (–C–O–C–), and hydroxyl (–OH) functional groups. Moreover, limitation of GO use in a variety of biomedical applications is due to the absence of stable dispersions. In the following sections, we review current nanotoxicity studies carried out with GO over the last 5 years. Moreover, Tables 1 and 2 provide a thorough summary of all of the current studies, which address functionalized GO cytotoxicity from in vitro and in vivo studies.

**Table 1:** Summary of in vitro study of functionalized graphene oxide toxicity reviewed

Functionalization	Cell line/animal model	Concentration and duration	Summary results	References
GO	A549	0, 10, 25, 50, 100, 200 μg/mL, 24 h	GO hardly enters cells and shows good biocompatibility, dose and size related	Chang et al. (2011a)
Chitosan-GO	MC3T3-E1 mouse pre-osteoblast cell line	CS-1 wt% GO, CS-3 wt% GO, 14 days	GO into a CS network favorably modulated the biological response of osteoblasts, such that cell attachment, proliferation, and growth were significantly enhanced	Depan et al. (2011)
GO/TiO_2_	HeLa	25, 50, 75, 100 μg/mL, 20 min	GOT caused antioxidant enzyme activities reduction and various apoptotic events in HeLa cell line, and induced apoptotic death	Hu et al. (2012)
GO/DOX gel	human nasopharyngeal carcinoma CNE1 cells	6 mg/mL GO 4, 2, 1 mg/mL DOX, 14 days	The gel exhibited good injectability, particularly in the case of higher amounts of GO or DOX. The in situ encapsulated DOX showed a sustained release behavior and antitumor efficacy	Ma et al. (2012)
HB-GO	HeLa, SMMC-7721, SGC-7901, A549	HB–GO (2:1), HB–GO (1:1)	The active uptake of HB–GO into tumor cells and significant damage to such impregnated cells was observed upon irradiation	Zhou et al. (2012b)
RGO/Gel	rabbit’s fibroblast cells	0.1, 0.3, 0.5, and 0.7 wt	RGO had no negative effect on cell growth, so the RGO/gel composite may be a promising biomaterial, with good cell compatibility	Wang et al. (2012)
MB/GO	DNA		The treated DNA increased the quenching efficiency of GO on MB compared to intact target DNA, indicating that all of them exert damage effect on DNA	Zhou et al. (2012a)
GO	Mice fibroblast cells line L929	100 μg/mL, 48 h	Materials show relatively good cyto-compatibility, the degree depends on the concentration and type of dispersant	Wojtoniszak et al. (2012)
LP-GO	HEK293 and HeLa cells	0.1 mg/mL	Efficiently condensed pDNA and delivered it to the insides of the cells. LP-GO-2 showed the capability to deliver siRNA efficiently into the cells	Tripathi et al. (2013)
GO	MG-63 cells	25, 50, 100, 200 μg/mL, 14 days	GO shows non-uniformity in size and shape of its particles and size variation hamper the transfection of nanocomposite into the cells	Deepachitra et al. (2013)
PEI-GO/PEI-GO–UCNP	MCF-7/Kun Ming Mouse	10, 20, 40, 60, 80 μg/mL, 48 h	Efficient, versatile PEI-GO–UCNP with up-conversion luminescence exhibited high drug loading efficiency and controlled release of DOX to kill cancer cells	Yan et al. (2013)
GO	Neuroblastoma SH-SY5Y cells	0, 0.4, 4, 40, 400 μg/mL, 24 h	With Vpr13-33, giving rise to the transition in conformation, morphology and dimension changes of aggregates, and reduced cytotoxicity of Vpr13-33	Zhang et al. (2013)
GO	Mouse skeletal myoblasts C2C12	1.5 mg/mL, 24 h	The enhanced cellular behavior on graphene derivatives was attributed to surface roughness and surface oxygen content that influences the adsorption of serum proteins	Ku and Park (2013)
GNPs on ITO	NE-4C neuroectodermal stem cells	275 mg/l	Very effective for in situ monitoring of the undifferentiated and differentiated state of stem cells	Kim et al. (2013)
GO	Mouse peritoneal macrophages	0, 1, 5, 10, 50 μg/mL, 24 h	Potential toxic mechanism of carbon nanomaterials and suggest caution on their utilization	Wan et al. (2013)
GO	PMEFs	0, 20, 40, 60, 80, 100 μg/mL, 24 h	M-rGO shows significant biocompatibility than GO at higher concentrations	Gurunathan et al. (2013b)
FA–NGO–PVP	Hela, A549	0, 30, 60, 100 μg/mL, 72 h	Cellular uptake demonstrated internalization of FA–NGO–PVP into tumor cells via receptor-mediated endocytosis and exhibited the cytotoxicity to Hela	Qin et al. (2013)
GO, LA-PEG-GO	HLF cells	1, 50, 100 μg/mL, 24 h	DNA damage induced by LA-PEG modified GO was mild compared with that induced by other GO derivatives	Wang et al. (2013a)
GO	*E. coli*	0, 25, 50, 75, 100, 125, 150 mg/mL, 4 h	Antibacterial activities are time and concentration dependent; the bacterial cell death may be due to oxidative stress and leads to DNA fragmentation	Gurunathan et al. (2013a)

**Table 2:** Summary of in vivo study of functionalized graphene oxide toxicity reviewed

Functionalization	Cell line/animal model	Concentration and duration	Summary results	References
^188^Re-GO	Male Kun Ming Mice 20 ± 2 g, 6–8 weeks	1 and 10 mg/mL	High values of %ID/g in urine within 12 h	Zhang et al. (2011b)
NGO-PEG-DOX	EMT6 cell/20 Balb/c female mice 6–8 weeks	24 μg/mL, 24 h/200 μL, 10 mg/kg, 7 days	Complete destruction of the tumors without weight loss or recurrence of tumors	Zhang et al. (2011a)
^66^Ga-NOTA-GO-TRC105	4T1, MCF-7, endothelial cells	50 μg/mL, 24 h	Tumor targeting of NOTA-GO-TRC105 was vasculature specific with little extravasation	Hong et al. (2012)
GO/pGO	5 weeks female Balb/c mice,7 weeks female C3H/HeN mice	10,20,40 μg/mL, 24 h	pGO accumulated to the tumor tissues, and systemic pGO nanoparticle-based co-delivery of Ce6 with DOX improved the efficacy of PDT	Miao et al. (2013)
GO-IONP-Au-PEG	4T1, human carcinoma KB cells	0. 0.625, 1.25, 2, 5, 5, 10 μg/mL, 18 days	Could serve as a photo-thermal agent for PTT cancer cell killing under molecular targeting or magnetic targeting shows excellent tumor ablation therapeutic efficacy	Shi et al. (2013b)
GO	Male athymic nude mice (CAnN.CgFoxn1nu/CrljOri, 6 weeks old)	0,10,25,50 μg/mL, 80 h	Pluronic-coated nanoGO efficiently showed an enhanced anticancer effect by combined PDT–PTT effect and exhibited high accumulation in tumor tissue	Sahu et al. (2013)

#### Functionalized graphene oxide toxicity in vitro

##### GO cytotoxicity

**Fig. 7:**
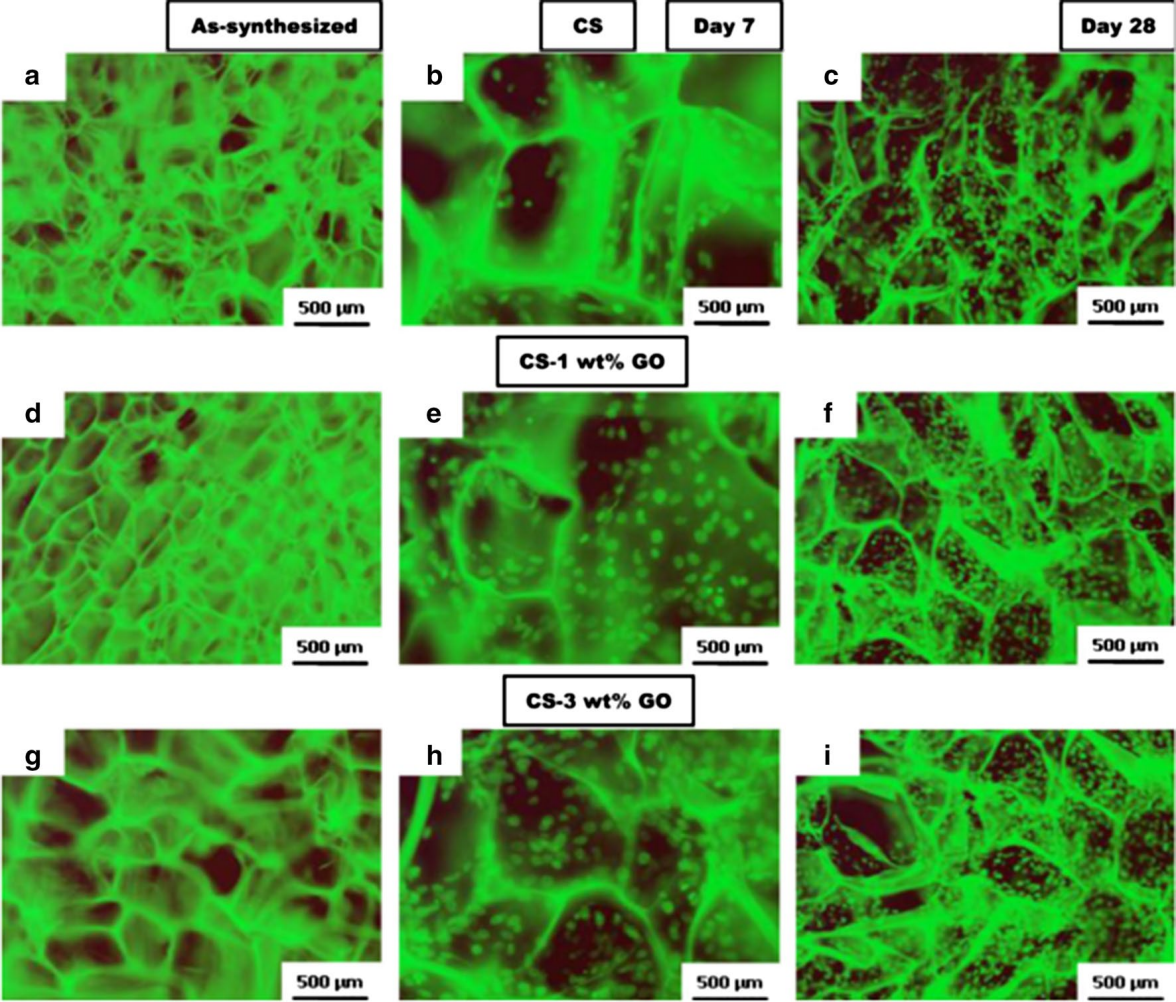
**a**–**i** Fluorescence micrographs illustrating the proliferation of pre-osteoblasts on pure CS and CS–GO scaffolds at similar locations (e.g., the center) after 7 and 28 days, respectively (Depan et al. 2011)

Initially, the influence of GO on the viability of A549 (human lung adenocarcinoma epithelial cell line) cells based on current data has shown that at low concentrations, GO does not enter into the cells and shows no signs of cytotoxicity. However, GO is known to be cytotoxic and is dose-dependent and known to cause oxidative stress in A549 cells, and induce a loss in cell viability at high concentrations (Chang et al. 2011a). Cell viability tests depict significant cell destruction by 1.0 μg/mL of reduced graphene oxide nanoplatelets (rGONPs) with average dimensions (ALDs) of 11 ± 4 nm, while the rGO sheets with ALDs of 3.8 ± 0.4 μm could show a significant cytotoxic effect only at high concentration of 100 μg/mL after 1-h exposure. Although oxidative stress and cell wall membrane damage were determined as the main mechanism involved in the cytotoxicity of the rGO sheets, neither of them could completely describe the cell destructions induced by the rGONPs, especially at the concentrations ≤1.0 μg/mL (Akhavan et al. 2012e). In other studies, microbially reduced graphene oxide (M-rGO) indicated the significant biocompatibility on primary mouse embryonic fibroblast (PMEF) at concentrations of 100 μg/ml (Gurunathan et al. 2013b). Furthermore, graphene chitosan synthesized by covalent linkage of carboxyl groups of GO with amine functionalized groups of chitosan was investigated. The negatively charged GO in chitosan scaffolds was an important physical and chemical factor, which enhanced cell scaffold interactions, as shown in Fig. 7 (Depan et al. 2011). Polyethylenimines (PEIs) are polymeric transfection agents, which are highly branched like and contains primary, secondary, and tertiary amino (–NH_2_) groups, whereas linear PEIs contain all secondary amines. The production of linear PEI-grafted GO (LP-GO) conjugates, and their efficacy to transfer nucleic acids into the mammalian cells is investigated (Tripathi et al. 2013). The LP-GOs interact with negatively charged nucleic acids and transport them efficiently into cells, therefore, indicating that the conjugates have high transfection efficiency and have better cell viability compared to LP (Tripathi et al. 2013). In other studies, cytotoxicity and genotoxicity data of GO to human lung fibroblast (HLF) cells have been assessed with the MTT assay, sub-G1 measurements, and comet assays (Wang et al. 2013a), and the results present concentration dependency. This study considered four different concentrations, 0, 1, 50, and 100 μg, and indicated better response to the higher concentration range. The cell response has been studied to synthesize lactobionic acid–polyethylene glycol (LA–PEG)-functionalized graphene oxide (LA–PEG–GO), PEG-functionalized graphene oxide (PEG–GO), PEI-functionalized graphene oxide (PEI–GO), and GO. The resulting cell was response revealed more positive to GO, PEI–GO, PEG–GO, and LA–PEG–GO, respectively. The genotoxicity induced by GO was more severe than the cytotoxicity to HLF cells. The toxic effect can be explained by the oxidative stress mediated by GO. In addition, the electric charge on the surface of GO is crucial having shown to decrease the toxicity of GO (Wang et al. 2013a). No toxicity was observed on endothelial cells (ECs) grown on PEI–GO–UNCP, and high potential of dead cancer cells on PEI–GO–UNCP was observed (Yan et al. 2013). In other studies, toxicity evaluation of acid-functionalized (Wan et al. 2013) GO induced autophagosome accumulation and the conversion of light chain 3 (LC3-I) to LC3-II (LC3 represents a mammalian homologue of the yeast autophagy-related gene ATG8). In addition, GO accumulation in macrophage lysosomes indicates the instability of lysosome membranes and leads to autophagic degradation (Wan et al. 2013). An investigation showed that GO was capable of stimulating myogenic differentiation and revealed myotube formation on GO (Ku and Park 2013). In this case, myogenic differentiation was significantly enhanced on GO base on the protein expression, formation, and expression of differentiation specific genes (MyoD, myogenin, Troponin T, and MHC). So the results indicated that the potential application for skeletal tissue engineering of GO is to stimulate myogenic differentiation (Ku and Park 2013). A further study investigated how PTT influenced cytotoxicity when using polyvinylpyrrolidone (PVP) functionalized GO (Qin et al. 2013). Here, folic acid (FA), a target molecule to cancer cells, was conjugated to GO via covalent –NH_2_ bonds, obtaining FA–NGO–PVP and then illustrating an ideal pH-responsive nanocarrier for delivery of an anticancer drug doxorubicin (DOX) with the loading ratio more than 100 % (Qin et al. 2013). In other studies, GO, titanium dioxide (TiO_2_) (GO/TiO_2_) hybrid (GOT) was studied by using Ti (OC_4_H_9_)_4_ and GO as reactants. The result presented no toxicity of GO in vitro as an electron sink in GOT efficiently increased the photodynamic therapy (PDT) activity (Hu et al. 2012). Furthermore, in vitro studies of fibrin-coated GO (FGO) indicated that high levels of alkaline phosphatase and calcium ion release lead to confirmation of osteo-inductive nature of FGO (Deepachitra et al. 2013), and MTT assay data showed the biocompatibility of osteoblast-like cell line MG-63 on GO. Furthermore, GO nanosheets used to induce in situ gelation of doxorubicin hydrochloride as an anti-tumor drug (Ma et al. 2012). Introduction of small amount of GO into aqueous solutions of doxorubicin hydrochloride caused the formation of thixotropic gel without any chemical additives (Ma et al. 2012). Cell growth confirmed that the materials remained cytocompatible with GO**-**based materials.

##### Genotoxicity of graphene-based nanomaterials

Investigations using nanoparticles less than 50 nm in each dimension, and GO with a lateral dimension of 2 μm and 1.5 nm in thickness at different concentrations were dependent factors in inducing genotoxicity, and graphene was found to cause the most damage to DNA (Qiao et al. 2013). A further study depicted DNA damage using nanoparticles of silicon dioxide (SiO_2_), ZnO, TiO_2_, tin (Sn), and CNTs at concentration higher than (100 μg/mL). Graphene concentrations higher than 1 μg/mL induced DNA damage at a significantly lower concentrations (Seabra et al. 2014; Qiao et al. 2013). Also the size-dependent genotoxic effects of rGO nanoplatelets (rGONPs) on mesenchymal stem cells (hMSCs) are investigated (Akhavan et al. 2012e). The rGONPs showed genotoxic effects on hMSCs through DNA fragmentation and chromosomal aberrations even at very low concentration of 0.1 mg/mL, highlighting concerns when using stem cells for applications for use in regenerative medicine.

##### Functionalized GO toxicity in vivo

**Fig. 8:**
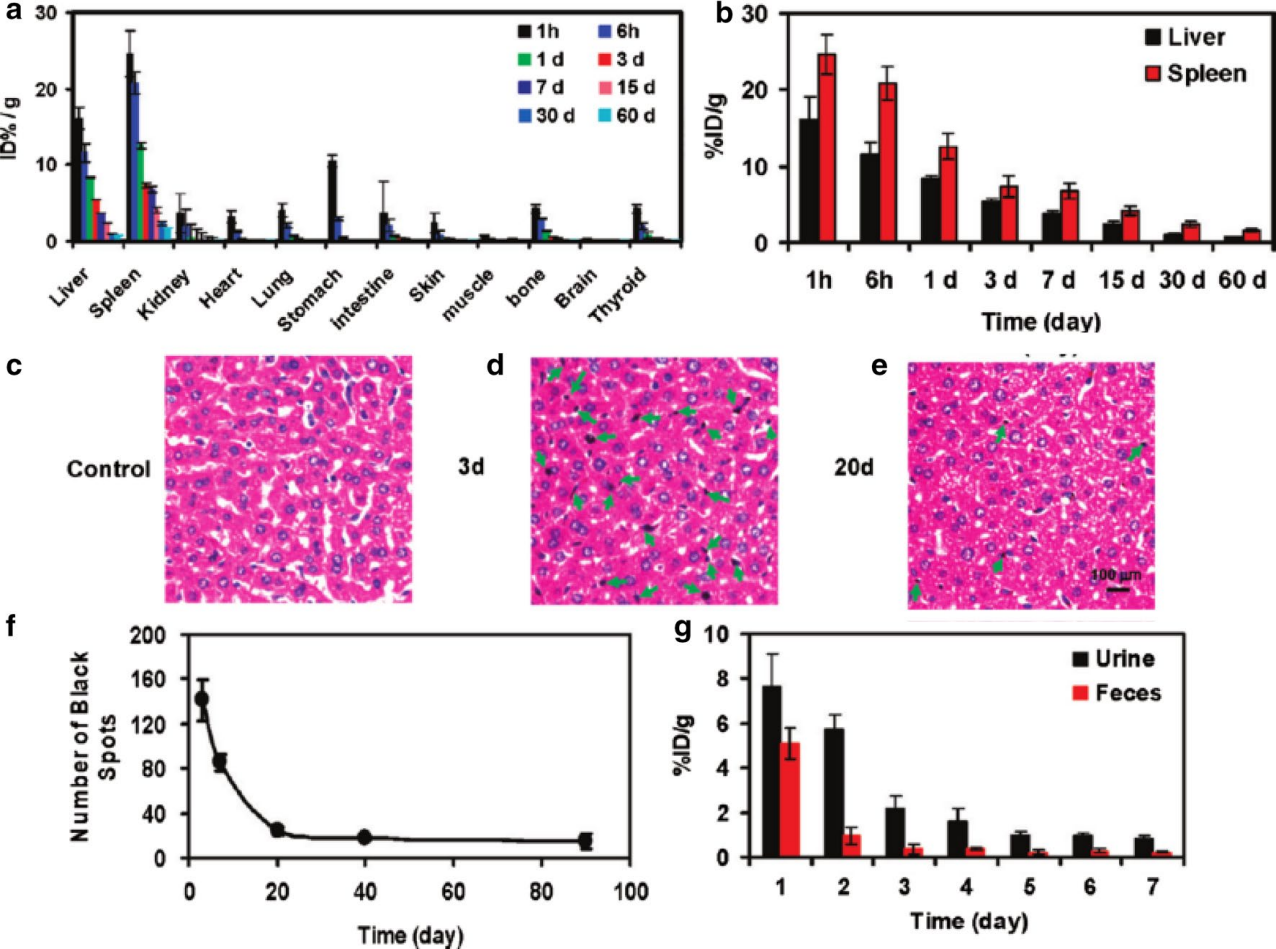
Biodistribution and clearance of NGS-PEG. **a** Time-dependent biodistribution of ^125^I-NGS-PEG in female Bal b/c mice. **b** ^125^I-NGS-PEG levels in the liver and spleen over time. **c**–**e** H&E stained liver slices from the untreated control mice (**c**) and NGS-PEG injected mice at 3 days (**d**) and 20 days (**e**) p.i. *Brown*-*black* spots which could be clearly differentiated from the blue-stained cell nuclei were noted in the liver of mice 3 days after injection of graphene. Much less black spots in the liver were observed 20 days later. **f** Statistic of *black spot numbers* in liver slices at various time post-injection of NGS-PEG. *Numbers* of spots in full image fields under a ×20 objective were averaged over 5 images at each data point. **g** ^125^I-NGS-PEG levels in urine and feces in the first week after injection. Mouse excretions were collected by metabolism cages. *Error bars* in the above data were based on standard deviations of 4–5 mice per group (Yang et al. 2011a, b)

Functionalized GO toxicity and their distribution have been studied in mice using radiolabeling techniques (Zhang et al. 2011b). Results indicate that GO has sufficient biocompatibility when studied in parallel with red blood cells (RBCs). In addition, GO mainly deposited in the lungs and surrounding tissue, and no pathological variation was illustrated when exposed to mice at 1 mg/kg body weight of GO for 14 days. But at a higher dosage, 10 mg/kg body weight, pulmonary edema, longtime retention, high accumulation, granuloma formation, inflammation, and cell infiltration was observed (Zhang et al. 2011b). Amino group termination covalently attached to GO via a six-arm branched glycol (PEG; 10 kDa) chains were conjugated to NOTA (1,4,7-triazacyclononane-1,4,7-triacetic acid, for ^66^Ga-labeling) and TRC105 (an antibody that binds to CD105) (Hong et al. 2012), and study of histology validated the characterization of the GO conjugates. The in vivo characterizations were performed in murine breast tumor mice (4T1), and great stability in mouse serum was exhibited in ^66^Ga-NOTA-GO and ^66^Ga-NOTA-GO-TRC105 conjugates. Quick accumulation of ^66^Ga-NOTA-GO-TRC105 in tumor uptake remained stable (Hong et al. 2012). In another study, GO functionalization by iron oxide nanoparticles (IONPs) and gold, forming a multi-functional magnetic and plasmonic GO-IONP-Au nanocomposite with strong super-paramagnetism, significantly enhanced optical absorbance in the NIR region (Shi et al. 2013b). Enhanced photo-thermal cancer ablation effect using GO-IONP-Au-PEG is realized in comparison with PEGylated GO used in earlier studies, as demonstrated in in vivo animal experiments. Moreover, the IONP and Au compartments in the GO-IONP-Au-PEG nanocomposite could prove to be advantageous for magnetic resonance (MR) and X-ray dual-modal imaging (Shi et al. 2013b). Non-covalently functionalized nanographene oxide sheet (nano-GO) with pluronic block copolymer and positively charged photosensitizers via electrostatic interactions have been previously reported (Sahu et al. 2013). These applications were combined with photodynamic thermal therapy (PDT) and PTT for cancer. Cancer cells show increased uptake when compared to normal cells by the use of the nano-GO, and it showed no toxicity to cells in the absence of NIR. High tumor accumulation was observed as a complex was injected intravenously into the tumor. Then, total ablation of tissue caused by NIR explosion via PDT and PTT (Sahu et al. 2013). In further studies, it was shown that doxorubicin loaded on to polyethylene glycol (PEG)ylated graphene oxide (GO–PEG–DOX) facilitates combined chemotherapy and PTT (Zhang et al. 2011a). The GO–PEG–DOX nanoparticle ability to combine local, site-specific chemotherapy with external near-infrared-photo-thermal therapy (NIR-PTT) significantly improved the therapeutic efficacy of cancer treatment. In addition, the pathologic examination of main organs improved, as their toxicity study showed less toxicity results with GO–PEG–DOX compared to DOX (Zhang et al. 2011a). Furthermore, injection of 80 mg/kg polyethylene glycol–grafted graphene oxide (PEG–GO) into mice intravenously was investigated (Miao et al. 2013) and demonstrated the enhancement of cellular delivery compared to chlorin e6 (Ce6), as a natural molecule, and a promising photosensitizer. Accumulation of Ce6/Dox/PEG–GO in tumor tissues is shown in molecular imaging of mice, and substantial disruption of tumor nuclei was observed (Miao et al. 2013). Furthermore, photosensitizer molecule, 2-(1-hexyloxyethyl)-2-devinyl pyropheophorbide-alpha (HPPH or Photochlor^®^) loaded onto PEG-functionalized graphene oxide (GO) via supramolecular π–π stacking investigated and obtained GO–PEG–HPPH complex, shows high HPPH loading efficiency. The in vivo distribution and delivery were tracked by fluorescence imaging as well as positron emission tomography (PET) after radiolabeling of HPPH with ^64^Cu. Compared with free HPPH, GO–PEG–HPPH offers dramatically improved photodynamic cancer cell killing efficacy due to the increased tumor delivery of HPPH (Rong et al. 2014). In vivo biodistribution, and potential toxicity of as-made GO and a number of polyethylene glycol (PEG)-functionalized GO derivatives with different sizes and surface coatings, after oral and intraperitoneal administration at high doses are investigated (Yang et al. 2013a). Insignificant tissue uptake via oral administration on ^125^I-labeled PEGylated GO derivatives is observed, indicating the rather limited intestinal adsorption of those nanomaterials. In contrast, PEGyalted GO derivatives highly accumulated, but not as-made GO, in the reticuloendothelial (RES) system including liver and spleen were observed post-injection (i.p.) and are highlighted in Fig. 8. Moreover, studies based on histological examination of organ slices and hematological analysis discovered that insignificant toxicity to the treated animals, although GO and PEGylated GO derivatives were retained in the mouse over a long period of time after post-injection (Yang et al. 2013a).

### Toxicity of functionalized graphene

Among the graphene-based materials, graphene due to its super hydrophobicity is potentially more toxic than GO. However, this can be lessened by functionalizing graphene with polar chemical groups, which could aid the water solubility of graphene. In the following sections, we review the toxicity studies based on functionalized graphene (represented in Table 3) to present a summary of in vitro functionalized graphene toxicity.

**Table 3:** Summary of in vitro study of functionalized graphene toxicity reviewed

Functionalization	Cell line/animal model	Concentration and duration	Summary results	References
G	human glioma cell line U251	2.5–10 μg/mL, 24 h	Better photo-thermal efficiency of graphene, due to dispersibility/smaller size of graphene, is superior to that of its structural sibling	Markovic et al. (2011)
PTCA/CCG	HeLa, MDA-MB-231, K562 cells, NIH3T3	100 μg/mL, 72 h	Apta-sensor has the ability to differentiate cancer cells and normal ones and can be regenerated using AS1411 cDNA and reusable for cancer cell detection	Feng et al. (2011)
G	Hippocampus	100 μg/mL, 7 days	Biocompatible and capable of promoting neurite sprouting and outgrowth, during the early developmental phase	Li et al. (2011)
PGE/Graphene	HRP/DNA	1 μg/mL, 24 h	Glycidamide could induce more serious DNA damage than acrylamide	Qiu et al. (2011)
G	Human hepatoma HepG2	1 μg/mL, 48 h	iTRAQ-coupled 2D LC–MS/MS proteome analysis is effective to the cellular functions in response to nanomaterials.	Yuan et al. (2011)
G/GO	Mouse iPSCs cell line 20D17	1.5 mg/mL, 9 days	Allow for attachment, proliferate on and differential differentiation of iPSCs and promise for iPSCs	Chen et al. (2012b)
G	RAW 264.7	5, 10, 20, 40, 80, 100 μg/mL, 48 h	Graphene induce cytotoxicity and increase intracellular reactive oxygen species, and then trigger apoptosis by activation of the mitochondrial pathway	Li et al. (2012)
rGONPs	hMSCs	0.01–100 μg/mL, 24 h	The size- and concentration-dependent cytotoxicity of the graphene oxide sheets and nanoplatelets in the hMSCs were studied	Akhavan et al. (2012d, e, 2013)
G	hESC lines H9 from WiCell	0, 10, 25, 50 g/l, 7 days	Neuronal differentiation circumvents cytotoxicity and may potentially be developed into 3D AC-collagen structures, further enhancing cellular functionalization	Chen et al. (2012a)
rGO/GO	A549, RAW 264.7	200 μg/mL, 5 days	An important parameter determining the biological effects of rGO/GO	Horváth et al. (2013)
N/graphene	L929 cell line, EAHY926 cell line	100 μg/mL, 7 days	The blood assays indicate that N/graphene has slightly lower platelet adhesion and prolonged kinetic blood-clotting time than pristine graphene	Guo et al. (2013)
O-GNR coated with PEG-DSPE	HeLa, mouse fibroblast cells, SKBR3, MCF7	10, 50, 100, 200, 300, 400 μg/mL, 48 h	The higher uptake indicates that O-GNR-PEG-DSPEs have a dose, and time-dependent, and differential cytotoxic effects on the four cell lines	Mullick Chowdhury et al. (2013)
G	T87	0, 40, 60 mg/l, 72 h	Graphene induced necrosis in T87 cells by interfering with the morphology, plasma membrane disturbances, and mitochondrial dysfunction	Begum and Fugetsu (2013)
rGO/QC-PEG	KB cancer cell line	0.5, 1, 10 μg/mL, 72 h	Due to introduction of Plu-SH, the created space between rGO/QC-PEG plate and Plu-SH polymer aids to entrap more DOX or QDs enabling to show more drug loading efficiency and fluorescence	Al-Nahain et al. (2013)
G/Nafion	HeLa	100 μg/mL, 24 h	Excellent electrochemical sensing capability with good sensitivity, linearity of response, and bioaffinity	Yoon et al. (2013a, b)
PLA/GNP	Mouse embryo fibroblasts 3T3 (ATCC CCL-164)	1, 5, 10 μg/mL, 72 h	No considerable variation in cell proliferation at the surface of the films was observed, except those containing GO after 24 h	Pinto et al. (2013)
G	PANC-1	G Film, 24 h	Hard corona on the surface of graphene substrates can evolve significantly as one passes from protein concentrations	Mao et al. (2013a)
G	HT29, SW48 tumor cell lines	3 mg/l, 20 min	Polyphenol groups attached to rGO during reduction process are well selective binding agents to cancer cell surfaces	Abdolahad et al. (2013)
Gelatin-GNS	A549	5, 10, 50, 100, 150, 200, 300 μg/mL, 48 h	Gelatin-GNS showed excellent dispersibility and stability in distilled water and various physiological solutions, also exhibited a high drug loading capacity of MTX	An et al. (2013)
CRGO-COOH	Mouse prostate cancer cell line, TRAMP-C1	25 μg/mL, 180 h	UniformLy dispersed thermo-sensitive CGN, which displayed high photo-thermal properties and reversible dramatic size reduction with temperature increase	Wang et al. (2013b)
G-PEG	Phagocytic cells	10 μg/mL	GNMs to improve their dispersion in aqueous solutions for biomedical applications	Yang et al. (2013b)
G	HeLa cells	3 mg/Ml	These results demonstrate the importance of size-dependent graphene nanoflake toxicity	Yoon et al. (2013a)

#### Functionalized graphene toxicity in vitro

##### Cytotoxicity of functionalized graphene

In vitro toxicity studies with graphene revealed better results compared to CNTs in inducing PTT to destroy the human glioma cells, U251 cell line (Markovic et al. 2011). This involved oxidative stress and mitochondrial membrane depolarization resulting in mixed apoptotic and necrotic cell death characterized by caspase activation/DNA fragmentation and cell membrane damage, respectively (Markovic et al. 2011). In a further study, isobaric tags were used for relative and absolute quantification (iTRAQ)-coupled 2D liquid chromatography–tandem mass spectrometry (LC–MS/MS) approach to analyze the treated protein profile change in human hepatoma cells (HepG2) with graphene. The results showed less toxicity for moderate variation of protein levels for the cells treated with graphene (Yuan et al. 2011). Mouse induced pluripotent stem cell (iPSCs) culture and spontaneous differentiation into ectodermal and mesodermal lineages supported by graphene was investigated (Chen et al. 2012b). Graphene surface illustrated similar cell proliferation and adhesion of iPSC compared to glass substrates. Cytotoxicity effect-reduced graphene oxide nanoplatelets (rGONPs) on the human mesenchymal stem cells (hMSCs) were investigated (Akhavan et al. 2012e). The cell viability measurement indicated cell destructions at 1.0 μg/mL rGONPs; in contrast, the rGO sheets at concentration of 100 μg/mL illustrated a significant cytotoxic effect (Akhavan et al. 2012e). A further investigation using a single-layer-reduced GO nanoribbons (rGONRs) produced via an oxidative unzipping of MWCNTs (Akhavan et al. 2013). The cell viability assay on hMSCs with concentration of 10 μg/mL rGONRs indicated significant cytotoxicity effects, while the rGO sheets showed similar cytotoxicity at concentration of 100 μg/mL. The results illustrate the penetration of rGONRs into the cells and DNA fragmentation, as well as, chromosomal aberrations at concentrations of 1.0 μg/mL (Akhavan et al. 2013). The toxicity of graphene on macrophages and epithelial cells was also investigated. The results indicate that the initial exposure to these materials is most prominent in respiratory system (Horváth et al. 2013). The interaction of nitrogen ion-implanted graphene (NGR) has been evaluated with mouse fibroblast cells and human endothelial cells, as well as in blood compatibility studies using rabbit blood (Guo et al. 2013). The results indicated the cell viability and proliferation improvement of cells cultured on NGR compared with cells cultured on pristine graphene. Lower platelet adhesion, prolonged kinetic blood-clotting time, and hemolytic rate (below 5 %) presented for NGR showed thrombo-resistance than pristine graphene (Guo et al. 2013). The cytotoxicity of oxidized graphene nanoribbons (O-GNRs) water soluble with PEG–DSPE (1,2-distearoyl-sn-glycero-3-phosphoethanolamine-*N*-[amino(polyethylene glycol)]) was investigated (Mullick Chowdhury et al. 2013). The assays were conducted on Sloan–Kettering breast cancer cells (SKBR3), Henrietta Lacks cells (HeLa) derived from cervical cancer tissue, Michigan cancer foundation-7 breast cancer cells (MCF7), and National Institute of Health 3T3 mouse fibroblast cells (NIH-3T3) (Mullick Chowdhury et al. 2013). All of the cells decrease in cell viability, as they represented a time-dependent (12–48 h) and dose-dependent (10–400 μg/mL) response. It was found that SKBR3 and MCF7 show a significantly lowered cytotoxicity compared to HeLa cells. The cells incubated at 10 μg/mL concentration were 100 % viable. As the concentration increased to 400 μg/mL, the cell viability decreased to ~78 % of cells. On the other hand, significant dead cells were observed for HeLa cells even at concentration 10 μg/mL. The results indicated the heterogeneous cytotoxicity of O-GNR–PEG–DSPEs, which possessed different cytotoxicity compared with chemically reduced graphene (Mullick Chowdhury et al. 2013). In a related study, composite poly (lactic acid) (PLA) and PLA film filled with graphene-based materials investigated the biocompatibility. Graphene concentration of 10 μg/mL was used for mouse embryo fibroblasts incubated with both fillers. The results illustrate concentrations of graphene, and GO may perform decreased in toxicity biomedical applications (Pinto et al. 2013). Study of methotrexate (MTX) attached to the gelatin graphene nanosheets (gelatin-GNS) through strong π–π stacking interactions was conducted (An et al. 2013), and depicted great ability as a drug release, and high drug loading capacity of MTX. Based on the cytotoxicity results on A549 cell, the gelatin-GNS showed non-toxic at specific concentration while the MTX-gelatin-GNS depicted biocompatibility (An et al. 2013). Another study showed that protein-based facile method for fabrication of nanosized, reduced graphene oxide (nano-rGO) with high stability and low cytotoxicity was also investigated (Sheng et al. 2013). Highly integrated constructed photo-acoustic/ultrasonic dual modality imaging and photo-thermal therapy platforms further demonstrated that the prepared nano-rGO can be used as ready-to-use theranostic agents for both photo-acoustic imaging and photo-thermal therapy without further surface modification. Intravenous administration of nano-rGO in tumor bearing mice showed rapid and significant photo-acoustic signal enhancement in the tumor region, indicating its excellence for passive targeting and photo-acoustic imaging. Meanwhile, using a continuous-wave near-infrared laser, cancer cells in vivo were efficiently ablated, due to the photo-thermal effect of nano-rGO (Sheng et al. 2013). Number of neurite on graphene after cell seeding were enhanced during 7 days compared with tissue culture polystyrene (TCP) substrates (Li et al. 2011). In addition, the growth-associated protein-43 (GAP-43) was examined. Based on the results, GAP-43 expression was significantly enhanced in graphene when compared to TCP. This could result in the boost of neurite sprouting and outgrowth (Li et al. 2011). In brief, the presence of graphene indicated in vitro biocompatibility with different cell lines based on previous investigations. In contrast, there are not many sufficient studies on in vivo biocompatibility, which needs more investigations in this emerging field. Moreover, by functionalizing graphene, as polymer filler, the surface topography changes, which causes an increase in roughness and surface energy to modify the wettability. The polar component of surface free energy increased with GO and decreased with graphene added to the polymeric matrix.

##### Genotoxicity studies

Hemin-graphene nanosheets (H-GNs) that are able to distinguish intact and damaged DNA and catalytic activity of hemin through graphene π–π interactions have successfully synthesized. Based on this, for detection of single-stranded DNA (ss-DNA) and damage DNA induced by chemicals such as styrene oxide (SO), NaAsO_2_, and physical radiation, such as UV radiation, a free-label colorimetric method was developed. This method could be used to evaluate the new compounds’ genotoxicity, the maximum limit of pesticide residue, and food additives, due to its simplicity, sensitivity, speed, and cost-effectiveness (Wei et al. 2013). Still, the molecular basis for in vivo graphene oxide (GO) toxicity is unclear. Caenorhabditis elegans has been used to investigate the microRNAs (miRNAs) control of GO toxicity. A total of 23 up-regulated and 8 down-regulated miRNAs in GO-exposed nematodes were identified with the aid of SOLiD sequencing. The miRNA mutants were confirmed by the functions of identified miRNAs in regulating the GO toxicity on lifespan. Furthermore, the evidence to raise a hypothesis that GO may reduce lifespan through influencing the functions of insulin/IGF signaling, TOR signaling, and germline signaling pathways controlled by miRNAs provided (Wu et al. 2014).

#### Functionalized graphene toxicity in vivo

**Fig. 9:**
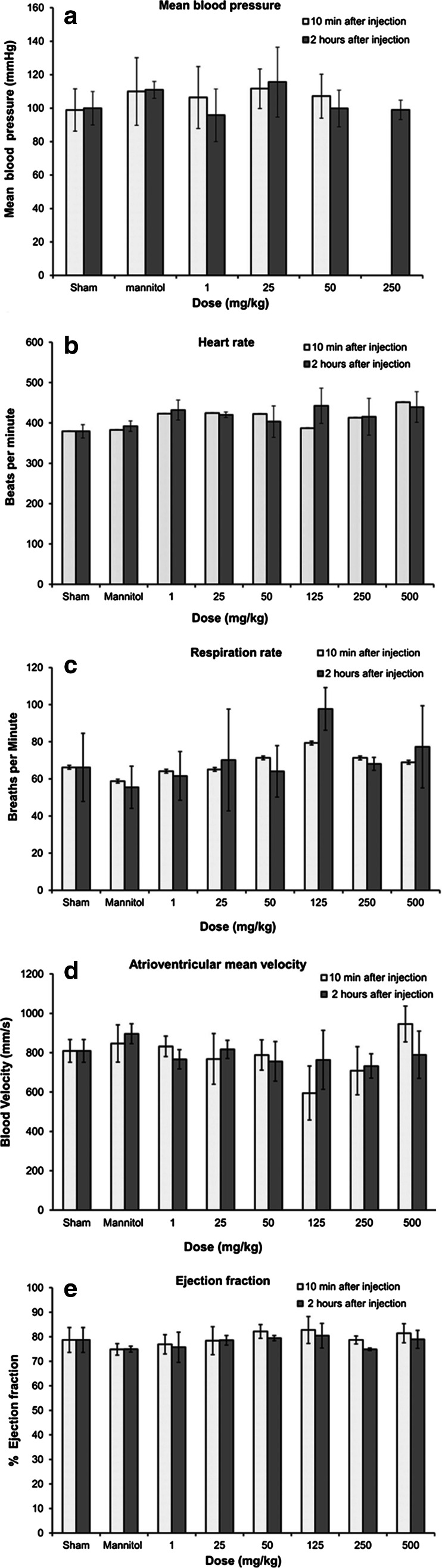
Hematological results from blood pressure and echocardiography measurements 10 min and 2 h post-injection of GNP-Dex (doses: 1–500 mg/kg). **a** Blood pressure, **b** heart rate, **c** respiration rate, **d** atrioventricular mean blood velocity, **e** % ejection fraction (Kanakia et al. 2014)

There are no sufficient in vivo studies addressing the toxicity of graphene based on PubMed database. One of the current ongoing research themes is regarding the magnetization procedure and in situ reduction, which used to convert GO on to magnetic graphene. This was modified covalently to construct poly-acrylic acid (PAA) for linking the fluorescein *o*-methacrylate (FMA) to yield multi-functional graphene (MFG) with water dispersibility based on 2.5 mg/mL concentration (Gollavelli and Ling 2012). In vivo studies with zebra fish indicated no effect on the survival rate after MFG microinjection, nor any significant abnormalities. Meanwhile, in vivo studies on HeLa cells presented that MFG is a biocompatible imaging probe with concentrations in the range of 100 μg/mL (Gollavelli and Ling 2012). Graphene nanoparticle dispersions indicated the multifunctional agents for in vivo biomedical applications. Therefore, regulatory guidelines for pharmaceuticals is followed, which recommend safety pharmacology assessment at least 10–100 times higher than the projected therapeutic dose and present comprehensive single dose response, expanded acute toxicology, toxico-kinetics, and cardiovascular pharmacology results for intravenously administered dextran-coated GO nanoplatelets (GNP-Dex) formulations to rats at doses between 1 and 500 mg/kg (Kanakia et al. 2014). The results presented that the maximum tolerable dose (MTD) of GNP-Dex is between 50 mg/kg ≤ MTD < 125 mg/kg blood half-life <30 min, and majority of nanoparticles excreted within 24 h through feces. Histopathology changes were noted at ≥250 mg/kg in the heart, liver, lung, spleen, and kidney; no changes in the brain and no GNP-Dex-related effects in the cardiovascular parameters or hematological factors were found (blood, lipid, and metabolic panels) at doses <125 mg/kg presented in Fig. 9. This result will open up new opportunities for pivotal preclinical single and repeat dose safety studies following good laboratory practices (GLP) as mandatory by regulatory agencies for investigational new drug (IND) applications (Kanakia et al. 2014).

## Conclusion

Graphene-based nanomaterials have great potential for large number of future technologies ranging from biotechnological and biomedical applications including, drug delivery, PTT, and cancer targeting and therapy. Graphene materials are known to vary widely in terms of their physical and chemical properties such as their dimensions and chemical functional groups. Moreover, they possess unique physical and chemical material properties such as optical, electrical and thermal conductivity, and high surface-to-volume ratio. In addition, they can be easily linked to macromolecules through covalent or non-covalent attachment. The types of graphene material and their chemical modifications to produce novel graphene compounds provide different levels of dispersibility and impurities within the nanomaterials. Based on the previous toxicity investigations of graphene, graphene oxide and their derivatives all exhibit in vitro toxicity. However, it showed that cells are very sensitive to size, shape, solubility, and concentration of graphene nanomaterials. GO is considered more biocompatible than graphene due to its greater solubility/dispersibility, which results in less damage and toxicity in human cell types such as skin fibroblasts and red blood cells, and bacteria. Unfortunately, there are no current or sufficient in vivo studies outlining their nanotoxicity. The results indicate that upon initial exposure of the materials, the most prominent route into the human body lies within the respiratory system. However, they are less effective in liver, kidney, and spleen. Furthermore, all cells exhibit time and dosage dependency depending upon protein adsorption and reactions governed by aggregation. As this review represents only a few studies in relation to graphene-based materials, their toxicology profile remains at the very early stages of development for a range of biomedical applications. However, before such materials reach the clinic, their toxicology profile and safety efficacies are essential steps in their evolution. Such great potential will offer a variety of new and powerful tools based on graphene materials for use in the areas of advanced imaging, disease diagnosis, and targeted therapies for the treatment of a range of severely debilitating diseases.

## Abbreviations

°C: Centigrade
0D: Zero dimensional
1D: One-dimensional
2D: Two-dimensional
3D: Three-dimensional
4T1: Murine breast tumor mice
A549: Human lung adenocarcinoma epithelial cell line
Au: Aurum, gold
CD105: Endoglin
Ce6: Chlorin e6
CGN: Thermo-sensitive nanogel
CNT: Carbon nanotube
CrGO: Chemically reduced GO
Cu: Copper
CVD: Chemical vapor deposition
Da: Dalton
DNA: Deoxyribonucleic acid
DOX: Doxorubicin
FA: Folic acid
FACS: Fluorescence-activated cell sorting
FDA: Food and drug administration
Fe_3_O_4_: Ferric oxide
FGO: Fibrin-coated GO
FMA: Fluorescein *O*-methacrylate
g: Gram
G: Graphene
Ga: Gallium
GAP-43: Growth-associated protein 43
Gel: Gelatin
Gelatin-GNS: Gelatin graphene nanosheets
GO: Graphene oxide
GOT: Graphene oxide/TiO_2_
HB: Hypocrellin B
HeLa: Henrietta Lacks cell
HepG2: Human hepatoma
HLF: Human lung fibroblast
hMSC: Human mesenchymal stem cells
HPPH: 2-(1-Hexyloxyethyl)-2-devinyl pyropheophorbide-alpha
IONP: Iron oxide nanoparticles
iPSC: Induced pluripotent stem cell
iTRAQ: Isobaric tags used for relative and absolute quantification
K: Kelvin
KClO_3_: Potassium chlorate
Kg: Kilogram
LA: Lactobionic acid
LC3: Light chain 3
LC–MS/MS: Liquid chromatography–tandem mass spectrometry
LP: Linear polyethylenimine
m: Meter
MCF7: Michigan cancer foundation-7 breast cancer cell
MFG: Multi-functional graphene
mg: Milligram
MG-63: Osteoblast-like cell line
MHC: Major histocompatibility complex
MHRA: Medicines and healthcare products regulatory agency
mL: Milliliter
MR: Magnetic resonance
M-rGO: Microbially reduced graphene oxide
MTT: Methyl thiazolyl tetrazolium
MTX: Methotrexate
MWNT: Multi-wall carbon nanotube
MyoD: Myogenin
NGR: Nitrogen ion-implanted graphene
Ni: Nickel
NIH-3T3: National institute of health 3T3 mouse fibroblast cell
NIR: Near-infrared
NOTA: 1,4,7-Triazacyclononane-1,4,7-triacetic acid
NSC: Neural stem cell
O-GNR: Oxidized graphene nanoribbons
PDT: Photodynamic thermal therapy
PEG: Polyethylene glycol
PEI: Polyethylenimine
PET: Positron emission tomography
PGE-DSPE: 1,2-Distearoyl-sn-glycero-3-phosphoethanolamine-*N*-[amino(polyethylene glycol)]
MCF7: Michigan cancer foundation-7 breast cancer cell
pH: Power of hydrogen
PLA: Polylactic acid
PMEF: Primary mouse embryonic fibroblast
PTT: Photo-thermal therapy
PVP: Polyvinylpyrrolidone
RBC: Red blood cell
rGO: Reduced graphene oxide
rGONP: Reduced graphene oxide nanoplatelet
RNA: Ribonucleic acid
SiC: Silicon carbide
SKBR3: Sloan–Kettering breast cancer cell
SWNT: Single-wall carbon nanotube
T: Troponin
TCP: Tissue culture polystyrene
Ti: Titanium
TiO_2_: Titanium dioxide
TPa: Tera pascal
TRC105: Human chimeric monoclonal antibody to CD105
U251: Human glioma cell
UCNP: Up-conversion nanoparticles
UK: United Kingdom
US: United States
W: Watt
μg: Microgram
